# Effectiveness of feeding supplementation in preterm infants: an overview of systematic reviews

**DOI:** 10.1186/s12887-021-03052-w

**Published:** 2022-01-04

**Authors:** Keqin Liu, Jiaxin Tao, Jixin Yang, Yufeng Li, Yanwei Su, Jing Mao

**Affiliations:** 1 School of Nursing, Tongji Medical College, Huazhong University of Science and Technology, Wuhan, 430030 Hubei China; 2 Department of Pediatric Surgery, Tongji Hospital, Tongji Medical College, Huazhong University of Science and Technology, Wuhan, 430030 China

**Keywords:** Preterm infants, Feeding supplementation, Nutritional feeding, Health outcomes

## Abstract

**Background:**

Preterm infants have higher nutrition needs than term infants. The effectiveness of various feeding supplementation was assessed by the improvement of health outcomes in single specific systematic reviews (SRs). The aim of this review was to comprehensively describe the effectiveness of feeding supplementation in promoting health outcomes of preterm infants.

**Methods:**

A literature search was conducted in the PUBMED, EMBASE, Science Direct, Cochrane library, Web of Science, and Wiley online library. SRs selection followed clear inclusion and exclusion criteria. Pairs of reviewers independently applied the criteria to both titles/abstracts and full texts. Screening and data extraction were performed by using the advanced tables. The methodological quality of SRs and the quality of the evidence were carried out according to the Assessing the Methodological Quality of Systematic Reviews (AMSTAR) tool and the Grades of Recommendation, Assessment, Development, and Evaluation guidelines (GRADE) respectively. A qualitative synthesis of evidence is presented.

**Results:**

Seventeen SRs were included in the review. Fifteen kinds of feeding supplementation were reported in the SRs. In preterm infants, the effectiveness of feeding supplementation in addition to regular breast-feeding was mainly shown in six aspects: physical health, neurodevelopment, biochemical outcomes, other health outcomes, morbidity and all-cause mortality. And the effectiveness of the interventions on health outcomes in preterm infants was found by most systematic reviews. The methodological quality of all the included SRs was high, and most of the evidences was of low or very low quality.

**Conclusions:**

Our results will allow a better understanding of the feeding supplementation in preterm infants. Although the feeling supplements may improve the health outcomes of in preterm infants, the existing evidence is uncertain. Therefore, the clinical use of these supplements should be considered cautiously and more well-designed RCTs are still needed to further address the unsolved problems of the included SRs.

## Background

Preterm infants, whose gestational age is less than 37 weeks, account for an increased risk of morbidity and mortality compared with term infants. [1] An estimated 15 million preterm infants are born each year, and the number is still increasing. [2] Premature delivery results in developmental delay; in addition, underdeveloped body systems make the preterm infant vulnerable to multiple diseases such as necrotizing enterocolitis, late-onset sepsis, and respiratory distress syndrome which are all major contributors to death. [3–5]

Sufficient nutrition support is essential for the growth and development of infants while preterm neonates have an even higher requirement for nutrition support. The adequate nutrient supply helps them to build up their bodies, boosts their immune system, and fights various infections. Generally, mothers’ milk has been proved to be the best food choice for neonates because it is full of nutritive and non-nutritive bioactive components. [6–8] Infant formula is also an effective substitute when breastfeeding is not available. [9] However, both of these might not meet the nutrition need of preterm infants considering their high demand. Extra supplementation of their feeding is apparently necessary.

Various kinds of feeding supplementation such as probiotics, lactoferrin and others have been tested in the primary studies, immaterial of whether they are RCTs or not, and some SRs have synthesised the relative evidence to assess the effects of supplementation. These SRs usually focussed on one particular supplement and different outcome assessment, which limited their ability to provide comprehensive evidence of the effectiveness of this feeding supplementation on growth improvement and disease prevention.

To the best assessment of the evidence from these SRs, this overview of systematic review was to comprehensively describe the effectiveness of feeding supplementation in promoting health outcomes of preterm infants.

## Methods

This review was drafted with reference to the Preferred Reporting Items for Systematic Reviews and Meta Analyses (PRISMA 2020) guidelines. [10] Given that all the data had been published online, no ethical approval or patient consent was required.

We used the PICO framework to develop the review question, which is based on describing the population (P), intervention s(I), comparison (C), and outcomes(O).

Population: Preterm infants (live babies born before completion of 37 weeks gestation), with a low or very low birth weight.

Intervention: Extra supplements were added to the milk to feed the preterm infants.

Comparison: Preterm infants were fed with breast milk or formula.

Outcome: A series of health outcomes were assessed. For example, physical growth, neurodevelopment, morbidity, and all-cause mortality.

Study design: Systematic reviews.

### Eligibility criteria

Inclusion criteria: (1) participants were preterm infants; (2) SRs focused on the comparison of nutrient supplementation to breast milk or formula in order to observe its effects on health improvement; (3) SRs contained a meta-analysis of the preterm infants’ outcomes and authors’ conclusions. When two or more similar SRs appeared on one topic, the year of publication, the number of primary included studies, an assessment of health outcomes, and quality of the content were thoroughly evaluated in order to determine the best choice for inclusion.

Exclusion criteria: (1) participants were not preterm infants; (2) in addition to systematic review, meta-analysis, or review; (3) intervention did not meet the inclusion criteria; (4) SRs not contain meta-analysis of the outcomes; (5) topic duplication.

### Information sources

A systematic search was conducted up to May 2020 in the following mainstream databases: PUBMED, EMBASE, Science Direct, Cochrane library, Web of Science, and Wiley online library. There was no language restriction applied. All references of included studies were reviewed and a series of health outcomes were assessed, looking for any additional systematic review that meets the inclusion criteria.

### Search strategies

We used population and intervention terms to develop search strategy: (‘preterm infant’ OR ‘premature infant’ OR ‘preterm neonate’ OR ‘low birth weight infant’ OR ‘very low birth weight infant’) AND (‘feeding supplementation’ OR ‘feeding supplement’ OR ‘supplement’ OR ‘feeding’). The article type was limited to systematic review, meta-analysis, or review.

### Selection process

Before the screening process, we pilot tested the screening tool in Microsoft Excel with the review team.

There were two screening stages. First, two reviewers independently screened for duplicate articles based on titles and abstracts. Secondly, following this first screened, two reviewers independently screened the full text review, excluded articles will be listed and given reasons for it. Subsequently, two reviewers independently applied the inclusion criteria using the standardized screening tool to both titles, abstracts and full texts. Disagreements were resolved by consensus. Discrepancies were discussed within pairs with a third reviewer if needed.

### Data collection process

For each eligible study, the data collection process took place in two steps. First, two reviewers collated the included studies and independently extracted the data. Secondly, a second reviewer validated all extracted data to ensure accuracy. Any discrepancies were resolved through discussion or discussed with a third reviewer.

### Data items

The data items onto each SR were extracted by using the predesigned data collection table including the following: the characteristics of the included studies which mainly contained the basic information of the research: author, year of publication, title, objective, study designs, number of included studies, sample size, type of participants, period of supplementation, and the characteristic of the interventions.

### Effect measures

Our study aims to describe the effectiveness of feeding supplementation in promoting health outcomes of preterm infants. Therefore, we looked for results such as improvement, effectiveness, hospital stay duration or other similar ones. Since different studies assessed different health outcomes and it was difficult to list them all in one small table, the review team discussed and agreed to classify the effectiveness of supplementation on health outcomes into six aspects in order to display: (1) physical growth: short term (less than 12 weeks), long term (at least 12 weeks); (2) neurodevelopment: short term (less than 12 weeks), long term (at least 12 weeks); (3) biochemical outcomes; (4) other health outcomes (time to achieve full enteral feeding, hospital stay duration etc.); (5) morbidity of any disease; (6) all-cause mortality.

### Synthesis methods

The synthesis process was according to PICO. It was based on feeding population, feeding materials and feeding effects that would allow a better understanding of feeding supplementation on health outcomes in preterm infants. To answer the question “what supplementation feeding have an impact on improving health outcomes of preterm infants”, the included studies were generated and implemented through discussion and consensus. Two reviewers followed the three steps by PICO form. First, extracted data were recorded by Microsoft Excel. The reviewers read the full texts of the included SRs and selected effective information. Second, the record was organized based on the various feeding supplementation. Thirdly, various feeding supplementation in the previous steps were grouped according to their effectiveness on health outcomes of preterm infants. The extracted data were converted to two tables: one reporting on the general characteristics of each review, and one reporting on characteristics of interventions.

### Quality assessment

The methodological quality of each SR was determined by the Assessing the Methodological Quality of Systematic Reviews (AMSTAR) tool. [11] There are 11 items on the tool and each item accounts for 1 point. The total score is 11 which means the highest quality of SR. The downgraded total score was categorized into 3 groups: 0–3, 4–7, and 8–11 which represented low, medium, and high quality. [12]

The evidences of outcomes which were of statistical significance, along with their quality were assessed by the Grades of Recommendation, Assessment, Development, and Evaluation guidelines (GRADE) [13]. The quality of the evidence was classified into four categories: high certainty: the true effect is close to the estimate of the effect; moderate certainty: the true effect is likely to be close to the estimate of the effect; low certainty: the true effect differs from the estimate of the effect; very low certainty: the true effect very differs from the estimate of the effect.

Two reviewers independently assessed the quality of each SR and any discrepancies was discussed and resolved by inviting, if necessary, a third reviewer to make the final decision. The quality of reporting was assessed according to the level of compliance with the PRISAM checklist (Supplementary file 1).

## Results

### Study selection

**Fig. 1:**
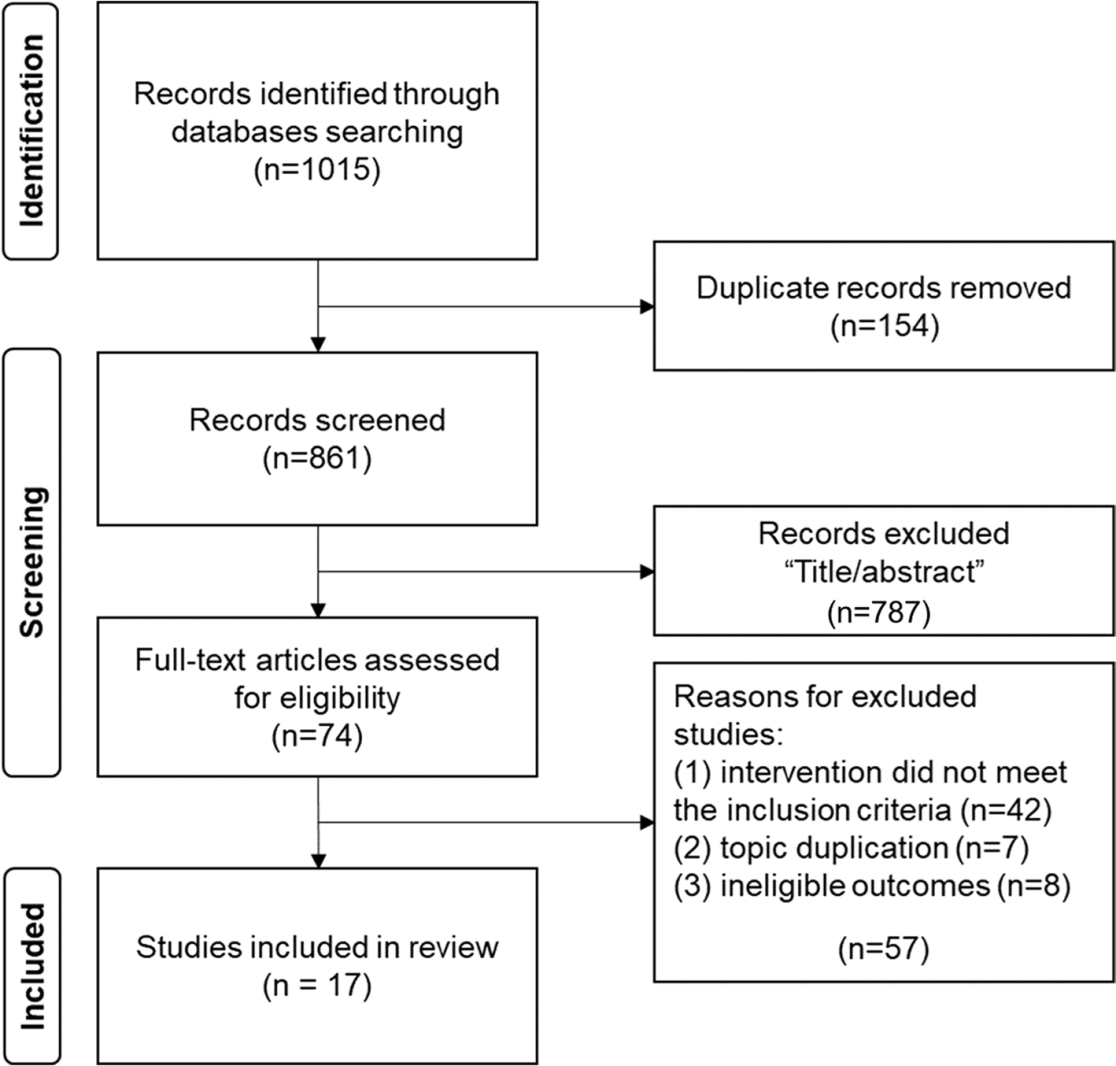
The flow diagram of study selection

The initial search for all the potential SRs from the databases returned 1015 records, of which 861 remained after removing duplicates. Through title screening and abstract reading, 74 records were kept for full-text reading assessment. There were further 57 articles excluded for the following reasons: (1) intervention did not meet the inclusion criteria; (2) topic duplication; and (3) there was no meta-analysis of the outcomes. Altogether, 17 eligible SRs met the inclusion criteria of this review. The study selection process is reflected in Fig. 1.

### Characteristic of included studies

In total, 161 quantitative primary studies were synthesized in the 17 SRs, all of which were RCTs or quasi-RCTs. Nine SRs [3, 14–21] were published in the late 3 years (2018–2020), 5 SRs [22–26] were between year 2016 to year 2017 and only 3 SRs [27–29] were from year 2007, year 2013 and year 2014. Fourteen SRs [3, 14, 15, 17, 19–23, 25–29] were Cochrane SRs and 3 SRs [16, 18, 24] were conventional ones. As for the number of studies included, only 6 SRs [14, 16, 24–27] incorporated more than 10 primary studies with a relatively larger sample size. Considering the different health outcome assessments, some SRs [16, 17, 24, 27] focused on the same interventions, and all were included to better observe the effect. The characteristics of all the included SRs are summarized in Table 1.

**Table 1:** Characteristic of the included studies

Author (Year)	Objectives	Study designs	NO. of studies/Sample size	Type of participants	Period of supplementation	Effectiveness of intervention	Overall quality of SRs
Pammi et al. (2020)	To assess the effect and safety of lactoferrin to prevent LOS and NEC in preterm infants	RCTs and quasi-RCTs	12/5425	GW<37w	NR	B, D, E, F	high
Howlett et al. (2019)	To assess the effectiveness and safety of supplementary inositol in preterm infants in reducing adverse neonatal outcomes.	RCTs and quasi-RCTs	3/1177	GW<37w and/or BW<2500 g	NR	E, F	high
Walsh et al. (2019)	To assess the evidence form RCTs that dietary supplementation with iodine reduces mortality and morbidity in preterm infants	RCTs or quasi-RCTs	2/1394	GW<37w	NICU stay	A, B, C, D, E, F	high
Chi et al. (2019)	To assess the effects of prebiptics in promoting health or preventing adverse health outcomes in preterm infants.	RCTs	18/1322	GW<37w or BW<2500 g	NR	D, E, F	high
Armannia et al. (2019)	To determine whether administration of prebiotics reduces the incidence of hyperbilirubinarmia among term and preterm infants.	RCTs or quasi-RCTs	3/154	(1) GW ≥37w (2)35w ≤ GW<37w (3)GW<37w	NICU stay	C, D, E, F	high
Amissah et al. (2018)	To determine whether supplementation of human milk with fat to preterm infants improve a series of health outcomes without adverse effects.	RCT	1/14	GW<37w	Hospital stays	A, D	high
Amissah et al. (2018)	To determine whether supplementation of human milk with protein to preterm infants improve a series of health outcomes without adverse effect.	RCTs or quasi-RCTs	6/204	GW<37w	Hospital stays	A, C, D, E	high
Amissah et al. (2018)	To determine whether supplementation of human milk with Carbohydrate to preterm infants improve a series of health outcomes without adverse effect.	Quasi-RCTs	1/75	GW<37w	Hospital stays	A, D, E	high
Yang et al. (2018)	To assess the effect of vitamin D on body development,immune function and disease prevention in preterm infants.	RCTs	12/NR	NR	Within 21d of birth	A, C, E	high
Harding et al. (2017)	To determine whether addition of calcium and phosphorus supplements to human milk leads to improved growth and bone metabolism of preterm infants	RCTs and quasi-RCTs	1/40	GW<37w	Hospital stays	A, C, D	high
Shah et al. (2017)	To exam the effect of arginine supplementation on the incidence of NEC in preterm infants.	RCTs and quasi-RCTs	3/285	GW<37w	NICU stay	A, B, E, F	high
Aceti et al. (2017)	To evaluate the effect of probiotics for LOS prevention in preterm infants.	RCTs	25/5868	GW<37w and/or BW<2500 g	NR	E	high
Moe-Byrne et al. (2016)	To determine the effects of gluta mine supplementation on mortality and morbidity in preterm infants.	RCTs and quasi-RCTs	12/2877	GW<37w	NR	A, B, D, E,F	high
Moon et al. (2016)	To assess whether supplementation of formula milk with LCPUFA is safe and of benefit to preterm infants.	RCTs	17/2260	GW<37w	NICU stay	A, B	high
AlFaleh et al. (2014)	To compare the efficacy and safety of prophylactic enteral probiotics administration in the prevention of NEC or sepsis in preterm infants.	RCTs or quasi-RCTs	24/5529	GW<37w and/or BW<2500 g	NR	A, B, D, E,F	high
Young et al. (2010)	To determine the effect of feeding preterm infants following hospital discharge with multi-nutrient fortified breast milk versus unfortified breast milk on growth and development.	RCTs or quasi-RCTs	2/246	GW<37w BW<2500 g	Hospital discharge	A, B, D	high
Verner et al.(2007)	To assess the effect of providing supplemental taurine for enterally or parenterally fed preterm or low birth weight infants on growth and development.	RCTs or quasi-RCTs	9/189	GW<37w BW<2500 g	Within 28d of birth	A, B, D, E,F	high

*NR* not reported; *GW* gestation week; *BW* birth weight

A: Physical health; B: Neurodevelopment; C: Biochemical outcomes; D: Other health outcomes; E: Morbidity of any disease; F: All-cause mortality

### Methodological quality of included SRs according to AMSTAR

The results of the quality assessment according to AMSTAR tool are shown in Table 2. All the SRs were of high quality indicated that the methodological quality of most of the included systematic reviews was generally of sufficient standard. 2 SRs [14, 26] scoring 11, 10 SRs [3, 15, 17, 20, 22, 24, 25, 27–29] scoring 10, 3 SRs [16, 19, 21] scoring 9 and 2 SRs [18, 23] scoring 8. All the SRs met the AMSTAR criteria 1 (prior design provided), criteria 2 (duplicate study selection and data extraction), criteria 6 (characteristics of the included studies provided), criteria 7 (scientific quality of the included studies assessed and documented), and criteria 8 (scientific quality of the included studies used appropriately in formulating conclusions). The least SR met AMSTAR criteria was 10 (likelihood of publication bias assessed) owing to the small number of the primary quantitative studies included in several SRs [3, 15, 17, 19–23, 25, 27–29].

**Table 2:** Methodological quality assessment of included studies according to the AMSTAR tool

Study	A	B	C	D	E	F	G	H	I	J	K	Total
Pammi et al.	1	1	1	1	1	1	1	1	1	1	1	11
Howlett et al.	1	1	1	1	1	1	1	1	1	0	1	10
Walsh et al.	1	1	1	1	1	1	1	1	1	0	1	10
Chi et al.	1	1	1	0	0	1	1	1	1	1	1	9
Armannia et al.	1	1	1	1	1	1	1	1	1	0	1	10
Amissah et al.	1	1	1	1	1	1	1	1	0	0	1	9
Amissah et al.	1	1	1	1	1	1	1	1	1	0	1	10
Amissah et al.	1	1	1	1	1	1	1	1	0	0	1	9
Yang et al.	1	1	0	1	0	1	1	1	1	1	0	8
Harding et al.	1	1	1	1	1	1	1	1	0	0	0	8
Shah et al.	1	1	1	1	1	1	1	1	1	0	1	10
Aceti et al.	1	1	1	0	1	1	1	1	1	1	1	10
Moe-Byrne et al.	1	1	1	1	1	1	1	1	1	1	1	11
Moon et al.	1	1	1	1	1	1	1	1	1	0	1	10
AlFaleh et al.	1	1	1	1	1	1	1	1	1	0	1	10
Young et al.	1	1	1	1	1	1	1	1	1	0	1	10
Verner et al.	1	1	1	1	1	1	1	1	1	0	1	10

1 = Yes. 0 = No/ Unclear/ Not applicable. A. Was an “a priori” design provided? B. Was there duplicate study selection and data extraction? C. Was a comprehensive literature search performed? D. Was the status of publication (i.e., grey literature) used as an inclusion criterion? E. Was a list of studies (included and excluded) provided? F. Were the characteristics of the included studies provided? G. Was the scientific quality of the included studies assessed and documented? H. Was the scientific quality of the included studies used appropriately in formulating conclusions? I. Were the methods used to combine the findings of studies appropriate? J. Was the likelihood of publication bias assessed? K. Were potential conflicts of interest included?

### Effectiveness of the interventions on health outcomes and quality of evidence

The effectiveness of the interventions on specific health outcomes of the included systematic reviews are presented in Table 3. Overall, the systematic reviews identified a total of 17 independent randomized trails investigating the use of feeding supplementation to promote growth development and reduce incidence of disease on preterm infants. A total of 15 kinds of different nutrient supplementation for the feeding of preterm infants were summarized in this review. The feeding supplementation could be classified into seven categories: protein (protein, lactoferrin), carbohydrate (prebiotic, inositol), fat (fat, LCPUFA), amino acid (arginine, glutamine), mineral (calcium), vitamin (vitamin D), and others (iodine, multi-nutrient, and probiotic).

**Table 3:** Characteristic of interventions

Author	Feeding supplementation	Outcome improvement							
		Physical growth		Neurodevelopment		Biochemical outcomes	Other health outcomes	Morbidity of any disease	All-cause mortality
		ST	LT	ST	LT				
Pammi et al.	Lactoferrin	NR	NR	NR	NSD	NR	Hospital stay ↓^c^ urinary tract infection ↓^c^	LOS ↓^c^ Fungal sepsis ↓^b^	NSD
Howlett et al.	Inositol	NR	NR	NR	NR	NR	NR	NSD	Neonatal death ↓^b^
Walsh et al.	Iodine	NSD	NR	NR	NSD	NSD	NSD	NSD	NSD
Chi et al.	Prebiotics	NR	NR	NR	NR	NR	Days achieve full enteral feeding↓^d^ Hospital stay ↓^d^ Stool frequency ↑^c^	Sepsis ↓^a^	↓^a^
Armannia et al.	Prebiotics	NR	NR	NR	NR	NSD	Phototherapy rate ↓^c^ Hospital stay ↓^c^ Stool frequency ↑^a^	Hyperbilirubina-emia ↓^c^	NSD
Amissah et al.	Fat	NSD	NR	NR	NR	NR	NSD	NR	NR
Amissah et al.	Protein	Weight ↑^c^ Length ↑^c^ HC ↑^c^	NR	NR	NR	Blood urea nitrogen ↑^c^	Hospital stay ↑^d^	NSD	NR
Amissah et al.	Carbohydrate	Weight ↑^d^	NR	NR	NR	NR	Hospital stay ↓^d^	NSD	NR
Yang et al.	Vitamin D	Length ↑^a^ HC ↑^a^	NR	NR	NR	Ig-A ↑^a^ Ig-G ↑^c^ IL-12 ↑^b^	NR	NSD	NR
Harding et al.	Calcium and/or phosphorus	NSD	NR	NR	NR	NSD	NSD	NR	NR
Shah et al.	Arginine	NR	NSD	NR	NSD	NR	NR	NEC ↓^b^	NSD (death related to NEC ↓^b^)
Aceti et al.	Probiotic	NR	NR	NR	NR	NR	NR	LOS ↓^a^	NR
Moe-Byrne et al.	glutamine	NSD	NR	NR	NSD	NR	Days achieve full enteral feeding ↓	NSD	NSD
Moon et al.	LCPUFA	Weight ↑^c^; Length ↑^c^.	NSD	NR	NSD	NR	NR	NR	NR
AlFaleh et al.	Probiotics	NSD	NR	NR	NSD	NR	Hospital stay ↓^d^ Days achieve full enteral feeding ↓^d^	NEC ↓^c^	↓^d^
Young et al.	Mult-inutrient	NR	NSD	NR	Visual acuity↑^c^	NR	Bone mineral content (4/12 months) ↑^c^	NR	NR
Verner et al.	Taurine	NSD	NR	NSD	NR	NR	Intestinal fat absorption ↑^c^	NSD	NSD

ST, Short term; LT, Long Term;

NR, Not reported; NSD, No statistical significance

^a^, High certainty; ^b^, Moderate certainty; ^c^, low certainty; ^d^, very low certainty;

↑, Increase; ↓, Decrease;

LCPUFA, long chain polyunsaturated fatty acid; HC, head circumference; LOS, late-onset sepsis; NEC, necrotizing enterocolitis

Indication:

NEC is defined as Bell’s stage ≥II. [13]

Hospital stay measured in days to discharge. [13]

LOS is defined as the presence of a positive blood or cerebrospinal fluid culture taken 72 h after birth. [23]

Hyperbilirubinaemia is defined as follows: [1] for term and late preterm neonates (GW ≥ 35w), total bilirubin (TB) level is eligible for phototherapy or as absolute TB level ≥ 15 mg/dL. [2] for preterm neonates (GW < 35w), TB level is eligible for phototherapy or as absolute TB level > 1% of body weight. [16]

Stool frequency is defined as total number of defecations recorded per day during intervention. [16]

Days achieve full enteral feeding is defined as days from birth to establish full enteral tube feeds (at least 150 ml/kg/day). [25]

Bone mineral content assessed by dual energy X-ray absorptiometry and clinical or radiological evidence of rickets on long-term follow-up. [27]

Regarding the overall effectiveness of feeding supplementation on health outcomes, fourteen SRs [3, 14, 16–18, 20–22, 24–29] concluded that 12 substances were associated with health improvement as a result of the extra supplementation in breast milk or formula. They were lactoferrin, inositol, prebiotics, protein, carbohydrate, vitamin D, arginine, glutamine, LCPUFA, multi-nutrient, taurine, and probiotics which were added to the feeding of preterm infants at a particular dose. Three SRs [15, 19, 23] reported no effect of the supplements of iodine, fat, calcium and phosphorus on the preterm infants due to there was no statistical significance.

Given that different outcomes were reported in each SR, as shown in Table 3, the specific outcomes improved by feeding supplementation were classified into six aspects: physical growth, neurodevelopment, biochemical outcome, other health outcomes, morbidity, and all-cause mortality. And the quality of statistically significant evidences according to GRADE rating was shown in Table 4.

**Table 4:** Evidence quality assessment of according to the GRADE guidelines

First author	Health outcomes	Study limitation	Indirectness	Publication bias	Imprecision	Inconsistency	Confidence
Pammi et al.	Hospital stay ↓	-1	0	0	0	-1	low
	urinary tract infection ↓	-1	0	0	0	-1	low
	Morbidity: LOS ↓	-1	0	0	0	-1	low
	Morbidity: Fungal sepsis ↓	-1	0	0	0	0	moderate
Howlett et al.	Mortality: Neonatal death ↓	0	0	0	0	-1	moderate
Chi et al.	Days achieve full enteral feeding↓	0	0	-1	0	-2	Very low
	Hospital stay ↓	0	0	-1	0	-2	Very low
	Stool frequency ↑	0	0	-1	-1	0	low
	Morbidity: Sepsis ↓	0	0	0	0	0	high
	mortality↓	0	0	0	0	0	high
Armannia et al.	Phototherapy rate ↓	-1	0	0	-1	0	low
	Hospital stay ↓	-1	0	0	-1	0	low
	Stool frequency ↑	0	0	0	0	0	high
	Morbidity: Hyperbilirubina-emia ↓	-1	0	0	-1	0	low
Amissah et al.	Weight ↑	-1	0	0	0	-1	low
	Length ↑	-1	0	0	0	-1	low
	HC↑	-1	0	0	0	-1	low
	Blood urea nitrogen ↑	-1	0	0	0	-1	low
	Hospital stay ↑	-1	0	0	-2	0	Very low
Amissah et al.	Weight ↑	-1	-1	0	-1	0	Very low
	Hospital stay ↓	-1	-1	0	-1	0	Very low
Yang et al.	Length ↑	0	0	0	0	0	high
	HC ↑	0	0	0	0	0	high
	Ig-A ↑	0	0	0	0	0	high
	Ig-G ↑	0	0	-1	-1	0	low
	IL-12 ↑	0	0	0	-1	0	moderate
Shah et al.	Morbidity: NEC ↓	0	0	-1	0	0	moderate
Aceti et al.	Morbidity: LOS ↓	0	0	0	0	0	high
Moe-Byrne et al.	Days achieve full enteral feeding ↓	0	0	-1	0	0	moderate
Moon et al.	Weight ↑	0	0	-1	0	-1	low
	Length ↑	0	0	-1	0	-1	low
AlFaleh et al.	Hospital stay ↓	-1	0	0	0	-2	Very low
	Days achieve full enteral feeding ↓	-1	0	-1	0	-2	Very low
	NEC ↓	-1	0	-1	0	0	low
	Mortality↓	-2	0	0	-1	0	Very low
Young et al.	Visual acuity↑	0	0	-1	-1	0	low
	Bone mineral content (4/12 months) ↑	0	0	-1	-1	0	low
Verner et al.	Intestinal fat absorption ↑	0	0	0	-1	-1	low

*LOS* Late-onset sepsis; *HC* Head circumference; *NEC* Necrotizing enterocolits

Study limitation: Downgraded two level to study risk of bias at two or more points. Downgraded one level to study risk of bias at one point

Indirectness: Downgraded one level due to a conclusion indirectly

Publication bias: Downgraded on level due to funnel plot is not symmetrical or fewer than nine studies were included

Imprecision: Downgraded one level due to uncertainty about precision.

Inconsistency: Downgraded one level due to included studies : 75%≤ I^2^ ≤100%; Downgraded two level due to included studies: 50≤ I^2^ < 75%

#### Physical growth

The effectiveness of feeding supplementation on physical growth was assessed by twelve SRs provided results of meta-analysis. [15, 18–23, 25–29]. All included SRs were deemed of high quality.

Four SRs [18, 20, 21, 25] concluded that there were effects to physical growth improvement as a result of the extra supplementation in breast milk or formula.

Yang et al. [18] concluded that high dose Vitamin D feeding on preterm infants, length gain and head circumference gain were increased at short term, There was a high certainty of evidence according to the GRADE rating. Amissah et al. [20] concluded that protein supplementation in preterm feeding promotes growth in a short period of time. There was a low certainty of evidence according to the GRADE rating. Amissah et al. [21] concluded that prebiotics supplementation of human milk may increase in weight at short term. There was a very low certainty of evidence according to the GRADE rating. Moon et al. [25] concluded that LCPUFA supplementation in preterm infants promote weigh gain and length gain. There was a low certainty of evidence according to the GRADE rating.

Eight SRs [15, 19, 22, 23, 26–29] concluded that additional feeding supplementation on preterm infants not definite had an influence.

#### Neurodevelopment

The effectiveness of feeding supplementation on neurodevelopment was assessed by eight SRs provided results of meta-analysis. [14, 15, 22, 25–29]. All included SRs were deemed of high quality.

In long term, the effectiveness of feeding supplements of Lactoferrin, Iodine, Arginine, glutamine, LCPUFA, Probiotics and taurine on neurodevelopment all reported no statistical significance. [14, 15, 22, 25–27] Meantime, in short term, the effectiveness of feeding supplements of taurine was no statistical significance [29].

However, Young et al. [28] concluded a promotion effect of mult-inutrient supplement on Visual acuity. There was a low certainty of evidence according to the GRADE rating.

#### Biochemical outcomes

The effectiveness of feeding supplementation on biochemical outcomes was assessed by five SRs provided results of meta-analysis [15, 17, 18, 20, 23]. All included SRs were deemed of high quality.

Two SRs [18, 20] concluded that nutritional supplements had an impact on biochemical outcomes. Yang et al. [18] concluded Vitamin D supplementation in preterm infants increased Ig-A, Ig-G and IL-12 levels. The level of evidence quality was high, low and moderate respectively according to the GRADE rating.

Amissah et al. [20] compared to those who received no additional protein, these infants observed blood urea nitrogen increased. There was a low certainty of evidence according to the GRADE rating.

Meanwhile, there were three SRs [15, 17, 23] concluded that the feeding supplementation of iodine, prebiotics, calcium and/or phosphorus was not effective on biochemical outcomes.

#### Other health outcomes

The effectiveness of feeding supplementation on other health outcomes was assessed by twelve SRs provided results of meta-analysis [14–17, 19–21, 23, 26–29]. All included SRs were deemed of high quality.

Nine SRs [14, 16, 17, 20, 21, 26–29] concluded that the additional feeding supplementation on preterm infants have an influence on other health outcomes. Pammi et al. [14] reported the length of hospital stay and urinary tract infection were both decreased. There was a low certainty of evidence according to the GRADE rating. Chi et al. [16] showed that the use of prebiotics with preterm infants reduced time to achieve full enteral feeding and hospital stay, and increased the stool frequency. The quality of evidence according to the GRADE rating were very low, very low and low certainty. Armannia et al. [17] reported that phototherapy rate and hospital stay were decreased by feeding supplementation with prebiotics. The quality of evidence according to the GRADE rating was both rated as low certainty. However, the feeding of prebiotics supplements on preterm infants resulted in an increased stool frequency. There was a high certainty of evidence according to the GRADE rating. Amissah et al. [20] considered that adding extra protein to human milk for preterm infants might increase hospital days. There was a very low certainty of evidence according to the GRADE rating. Amissah et al. [21] found that the carbohydrate supplementation of human milk in preterm infants could reduce the length of hospital stay. There was a very low certainty of evidence according to the GRADE rating. Moe-Byrne et al. [26] concluded that glutamine supplementation could reduce the time to reach full enteral nutrition with moderate evidence quality reported. AlFaleh et al. [27] pooled the studies that probiotics significantly shorted the hospital days and showed a significant reduction in time to reach full enteral feeds. There was both very low certainty of evidences according to the GRADE rating. Young et al. [28] reported that after four or 12 months supplementation of mult-inutrient, the bone mineral contest was increased. There was a low certainty of evidence according to the GRADE rating. And Verner et al. [29] demonstrated that taurine might help infants fat absorption form the gastrointestinal tract. There was a low certainty of evidence according to the GRADE rating.

Meanwhile, some SRs [15, 19, 23] reported that feeding supplementation applied to preterm infants was not effective on other health outcomes.

#### Morbidity of disease

Thirteen SRs [3, 14–18, 20–22, 24, 26, 27, 29] were assessed by the effect of feeding supplements on of disease prevention. All included SRs were deemed of high quality.

Six SRs [14, 16, 17, 22, 24, 27] concluded that feeding supplementation applied to preterm infants had an impact on disease morbidity. Pammi et al. [14] suggested that lactoferrin supplementation of enteral feeds decreased late-onset sepsis. There was a low certainty of evidence according to the GRADE rating. Pammi et al. also reported that lactoferrin supplementation on preterm infants could reduce the morbidity of fungal sepsis. There was a moderate certainty of evidence according to the GRADE rating. Chi et al. [16] showed that the use of prebiotics with preterm infants could decrease the incidence of sepsis. There was a high certainty of evidence according to the GRADE rating. Armanian et al. [17] reported that probiotic feeding reduced the incidence of hyperbilirubinaemia. There was a low certainty of evidence according to the GRADE rating. Shah et al. [22] concluded that the feeding supplementation of ariginine could reduce the morbidity of NEC. There was a moderate certainty of evidence according to the GRADE rating. Aceti et al. [24] showed that probiotics supplementation could resulted in a significantly lower incidence of LOS. There was a high certainty of evidence according to the GRADE rating. And AlFaleh et al. [27] concluded that enteral supplementation of probiotics could prevent severe NEC in preterm infants. There was a low certainty of evidence according to the GRADE rating.

However, the current available reviews [3, 15, 18, 20, 21, 26, 29] indicated that the feeding supplementation of inositol, iodine, protein, carbohydrate, Vitamin D, glutamine and taurine showed no effect on disease morbidity of preterm infants.

#### All-cause mortality

Eight SRs [3, 15–17, 22, 26, 27, 29] included data to perform the meta-analysis for the effect of feeding supplements on morbidity. All included SRs were deemed of high quality.

Three SRs [3, 16, 27] concluded that feeding supplementation applied to preterm infants have an impact on morbidity. Howlett et al. [3] revealed that there was reduction in neonatal death with inositol supplementation. There was a moderate certainty of evidence according to the GRADE rating. Chi et al. [16] showed that the use of probiotics with preterm infants decreased the mortality. There was a high certainty of evidence according to the GRADE rating. And AlFaleh et al. [27] concluded that enteral supplementation of probiotics prevented all-cause mortality in preterm infants. There was a very low certainty of evidence according to the GRADE rating.

Five SRs [15, 17, 22, 26, 29] concluded that the additional feeding supplementation of iodine, prebiotics, arginine, glutamine and taurine had no influence on all-cause mortality. Among them, although Shah et al. [22] also indicated no effect of arginine on mortality, but the death related to NEC was reported to be reduced. There was a moderate certainty of evidence according to the GRADE rating.

## Discussion

### Summary of main findings

Although medical technology has made great progress in recent years, premature is still a global health problem which requires a substantial financial investment in order to save lives. [30] Furthermore, surviving preterm infants suffer from a higher rate of morbidity and mortality, as well as delayed physical and neurological development compared with term infants. [30] Because of all these potential complications, multiple preventative strategies have been developed and examined, and feeding supplementation is one of the nutrition methods that can alleviate the situation.

This review of the feeding supplementation of preterm infants gives an overall insight into the effectiveness of feeling supplementation on health improvement or disease prevention. In total, 15 kinds of nutrient supplementation were reported in the included SRs. And of those, 12 nutritional supplements were concluded to have effect on health outcomes, mainly in the following six aspects: physical growth, neurodevelopment, biochemical outcome, other health outcomes, morbidity, and all-cause mortality. Three feeding supplements reported in three SRs were concluded as having no statistically significant effect on health outcomes of preterm infants. However, although the methodological quality of included SRs was high, the quality of most evidences was of low to very low certainty.

The interventions reported to have health improvement were: lactoferrin, inositol, prebiotics, protein, carbohydrate, vitamin D, arginine, glutamine, LCPUFA, multi-nutrient, taurine, and probiotics. All the SRs were evaluated as being of high quality, with 5 SRs [14, 16, 23, 24, 27] included more than 10 RCTs and involved a relatively large number of participants, which guaranteed academic authority. In despite these advantages, implications or suggestions provided by the SRs included should not be ignored either.

Lactoferrin is the dominant protein in human milk and has been proved to perform a series of functions including a wide spectrum of anti-microbial effect, the immunomodulation of host defence, and the promotion of gut growth and maturation. [31] Though previous studies have confirmed the safety and effectiveness of the use of lactoferrin on humans, it is still hard to recommend it for clinical use in view of the publication bias and small studies of poor methodology expanding the effect size. [14] Probiotic refers to live strains of microorganisms while prebiotic refers to a nonviable food component. [32] Both of them could modulate the intestinal microbiota. [33] Chi et al. [15], Aceti et al. [24] and AlFaleh et al. [27] have reported the effect of prebiotics and probiotics supplementation on the health outcomes in the SRs respectively. The combination of probiotics and prebiotics might better exert synergistic effects in the intestinal microbiota management which could be a research point in the future. In another study, Amissah et al. [20] assessed the effect of protein supplementation on growth development, but the current evidence is still of low quality and the evidence is lacking of long term benefits or harms. It has been proved that arginine was the essential amino acid for infants, and the occurrence of NEC was associated with the low level of circulating arginine [34] Although the SR conducted by Shah et al. [22] confirmed its effect in preventing NEC, a larger sample size and more research details focused on the NEC stage 2 or 3 are still required. The daily intake of vitamin D for preterm infants recommended by the European Society of Paediatric Gastroenterology (ESPGAN) was 800–1000 IU/day. [35] The preterm infants in the high dose group (800–1000 IU/day) had better growth while the circulating concentrations of 25(OH) D, calcium, and phosphorus were found to have no statistical significance between the high dose group (800–1000 IU/day) and low dose group (400 IU/day). [18] Therefore, caution is still needed in adopting the optimal dose intake for preterm infants and we should not just follow the recommendations without consideration in case of the toxic effect.

The effectiveness of above nutrients supplementation was mainly characterized by promoting immune regulation and intestinal development, thereby promoting growth and reducing disease incidence. The conclusions in the included SRs about supplementations that had no impact or only had effect on other health outcomes are also important. First, no further research priority should be given to iodine, fat, calcium and phosphorus supplementation and the feeding of them to preterm infants. [15, 19, 23] Second, some nutrient supplementation, such as inositol, glutamine, multi-nutrient, and taurine might work as fortifying components of human milk, thus aiding the understanding of the nutritional requirements of preterm infants. [26, 28, 29] Third, research on the supplementation of prebiotic (for prevention of hyperbilirubinaemia), taurine, glutamine and multi-nutrient could focus on the feeding details including the dose and duration. [17, 26, 28, 29] Next, more well-designed RCTs are needed to address the unsolved issues in the SRs. Last but not least, all feeding supplementation for preterm infants should respect their parents’ choice, follow the ethical rules, and be proceeded with cautiously.

### Implications for practice and future researcher

To the best of our knowledge, this is the first comprehensive overview of SRs focusing on feeding supplementation in preterm infants. The summary of the characteristics of the SRs and the interventions can help researchers become quickly familiar with trends in this area, which might stimulate new ideas among them. Most of the included SRs were from Cochrane; they were of the highest methodological quality and will be updated every several years. [36] The strength of this review also lies in aspects including a systematic literature search, clear inclusion and exclusion criteria, standard data extraction, and a professional-quality assessment tool, making the results more robust and reliable.

### Limitations and evidence gap in this area

Limitations of the review were also inevitable. Most of the SRs included did not report the long-term physical growth and neurodevelopment outcome assessment. The quality of the evidence in most SRs was low to very low, thus requiring larger and more well-designed RCTs to raise quality. Although no language restriction was applied, all the included SRs were published in English which might have left some significant SRs uncaptured. The overlapping effect might also exist and we could not assess it due to the primary studies being included in more than one SR. [37]

## Conclusions

This overview of systematic reviews comprehensively describes the effectiveness of feeding supplementation in promoting health outcomes of preterm infants. Current evidence shows that supplementation of lactoferrin, inositol, prebiotics, protein, carbohydrate, vitamin D, arginine, glutamine, LCPUFA, multi-nutrient, taurine, and probiotics to the feeding of preterm infants could have an effect on health improvement. They are considerable to be clinical used but still needed to be cautious, especially those with a low and very low quality of evidence. In the future, more well-designed RCTs are still needed to further address the unsolved problems of the included SRs.

## Data Availability

The datasets used and/or analysed during the current study are available from the corresponding author on reasonable request.

## Abbreviations

SR: Systematic review
AMSTAR: Assessing the Methodological Quality of Systematic Reviews
PRISMA: Preferred Reporting Items for Systematic Reviews and Meta Analyses
GRADE: Grades of Recommendation, Assessment, Development, and Evaluation
ESPGAN: European Society of Paediatric Gastroenterology
NR: Not reported
ST: Short term
LT: Long Term
NSD: No statistical significance
LCPUFA: Long chain polyunsaturated fatty acid
HC: Head circumference
LOS: Late-onset sepsis
NEC: Necrotizing enterocolitis
GW: Gestation week
BW: Birth weight
